# Light convolutional neural network by neural architecture search and model pruning for bearing fault diagnosis and remaining useful life prediction

**DOI:** 10.1038/s41598-023-31532-9

**Published:** 2023-04-04

**Authors:** Diwang Ruan, Jinzhao Han, Jianping Yan, Clemens Gühmann

**Affiliations:** 1 School of Electrical Engineering and Computer Science, TU Berlin, Berlin, 10587 Germany; 2 Chair of Electronic Measurement and Diagnostic Technology, TU Berlin, Berlin, 10587 Germany; 3 School of Aeronautics and Astronautics, Zhejiang University, Hangzhou, 310027 China

**Keywords:** Electrical and electronic engineering, Mechanical engineering

## Abstract

Convolutional Neural Network (CNN) has been extensively used in bearing fault diagnosis and Remaining Useful Life (RUL) prediction. However, accompanied by CNN’s increasing performance is a deeper network structure and growing parameter size. This prevents it from being deployed in industrial applications with limited computation resources. To this end, this paper proposed a two-step method to build a cell-based light CNN by Neural Architecture Search (NAS) and weights-ranking-based model pruning. In the first step, a cell-based CNN was constructed with searched optimal cells and the number of stacking cells was limited to reduce the network size after influence analysis. To search for the optimal cells, a base CNN model with stacking cells was initially built, and Differentiable Architecture Search was adopted after continuous relaxation. In the second step, the connections in the built cell-based CNN were further reduced by weights-ranking-based pruning. Experiment data from the Case Western Reserve University was used for validation under the task of fault classification. Results showed that the CNN with only two cells achieved a test accuracy of 99.969% and kept at 99.968% even if 50% connections were removed. Furthermore, compared with base CNN, the parameter size of the 2-cells CNN was reduced from 9.677MB to 0.197MB. Finally, after minor revision, the network structure was adapted to achieve bearing RUL prediction and validated with the PRONOSTIA test data. Both tasks confirmed the feasibility and superiority of constructing a light cell-based CNN with NAS and pruning, which laid the potential to realize a light CNN in embedded systems.

## Introduction

With the rapid development of modern industries, there is an increasing demand for higher safety and reliability of mechanical systems. As a promising approach to meet the above demands, Prognostic and Health Management (PHM) technology has been receiving increasing research attention in recent years^1^. As a fundamental support component in rotating machines, the rolling bearings’ performance directly affects the equipment’s reliability. Its failures may result in enormous damage, economic loss and human safety. Therefore, reliable fault diagnosis and Remaining Useful Life (RUL) estimation for predictive maintenance of bearings are meaningful and practical. Machine learning and deep learning^2,3^ as typical data-driven methods for PHM have been attracting growing attention from academia and industry. CNN is the most widely used among various deep learning networks due to its powerful ability in feature extraction and complex representation learning, with many satisfying results achieved in bearing fault classification^4–8^ and in RUL prediction^9–13^.

Despite the promising performance in fault diagnosis and prognosis, these reported CNNs considerably depend on complex architectures and a large number of parameters usually determined empirically and long-time training. For example, the parameters of filters and the sequences of layers are set based on expert experience or trials. Furthermore, the model parameter size has increased as deeper and more complex models are applied to achieve better results. It entails that fully training all the parameters in these networks has become more inefficient and time-consuming than ever. Furthermore, with the advent of intelligent applications in embedded devices, lightweight networks have become highly-calling demand from industry for deployment in limited-resource environments^14^.

Regarding lightweight CNN design, there are two main directions, light network structure design and model compression. In terms of the former, there are two main kinds of methods, manual design and NAS-based design. For manual design, the basic idea is to replace the general convolution layer with depthwise separable convolution or group convolution, such as the typical lightweight CNNs, MobileNet and ShuffleNet. This method is easy and efficient. However, it highly depends on four basic operations (depthwise, pointwise, group, shuffle) and involves too much human experience. In contrast, Neural Architecture Search (NAS)^15^ is a technique for automatically generating a neural network architecture and is presently widely used in machine learning and deep learning. In some specific tasks like image classification, the network searched by NAS already has a comparable or even better performance than the current state-of-the-art manually designed ones^16–20^. For NAS-based design, the procedure is to search for the optimal network structure within defined discrete structure and operation sets. This means an optimization issue on discrete sets that are non-differentiable, which is usually solved by reinforcement learning or evolutionary algorithm, bringing low solving efficiency. To address this problem, the Differentiable Architecture Search (DARTS) was proposed by Liu et al.^21^, which converts the discrete operations into a continuous space by identifying the operation selection with continuous probability represented by a softmax function. Then the model architecture can be optimized by gradient descent, accelerating the searching speed of optimal light structure. To further reduce the network architecture search space, Zoph and Le^17^ defined a minimum architecture called a cell. It consists of nodes and computation operations and works as a micro CNN. Xie and Yuille^22^ limited the number of nodes in a cell to reduce the size of the entire search space. After determining the best cell architecture, one can stack the cells into a deeper network. In this way, the network architecture space will be dramatically reduced, and the task of learning the whole network architecture reduces to learning the cell architecture.

Besides lightweight structure design, model compression is another efficient method to build light CNN or compress existing networks. As to network compression, there are two main directions: AutoML-based and rule-based compressions. For the former, the preferred method is reinforcement learning, which is time-consuming and unexplainable, not feasible in engineering applications. For the latter, though there are five widely-used technologies, like weights sharing, weights pruning, quantification, knowledge distillation, and low-rank decomposition, pruning is the most widely used due to its simplicity and efficiency. The idea of pruning is to remove the less important neurons or operations and obtain a smaller and faster network. For example, 90% of the weights in VGG16 are from the fully connected layers but account for only 1% of the total floating-point operations^23^. Works presented in^24^ and^25^ use an iterative threshold-based pruning to learn the weights and simultaneously prune the unimportant ones. Authors of^26^ go for a more structured way by pruning at the filter and group levels.

As introduced above, the methodology of lightweight CNN structure design and model compression have been studied. However, in published works, these two directions were studied and applied individually, while the combination of lightweight structure design and model pruning to develop a light CNN is rarely addressed. Theoretically, these two methods can design light CNN from different aspects, and the combination of them will lead to a lightweight CNN to the maximum extent. This motivates the study of this work. In addition, regarding the application, very few researches address the lightweight CNN involved in bearing fault diagnosis and RUL prediction. To bridge the gap, this paper proposes a two-step hierarchical method with DARTS-based NAS and model pruning to explore a light CNN for fault classification and RUL prediction. Firstly, the search space is defined, representing all possible operations such as convolution, separable convolution, dilated convolution, max pooling and average pooling. Then, CNN is trained on the training dataset, and the weights of connections in cells and the weights of filters are both trained by back propagation. The optimal cell structure is extracted after training and used to build the optimal CNN. Furthermore, the model is pruned by removing unimportant connections to reduce computation demand. The most unimportant connections and operations are selected after ordering their importance by the corresponding contribution to the accuracy or loss. Finally, this paper adopts the cell-based CNN model to realize bearing fault diagnosis and then adapts it to achieve RUL prediction after small modification, which means the two main tasks in PHM can be addressed with the light CNN obtained by NAS and pruning.

The main contributions of this paper can be summarized as follows:

- With DARTS-based NAS and model pruning, a two-step hierarchical method is proposed to construct lightweight CNN, including network structure optimization and parameter pruning. The former reduces network size from the whole by searching optimal cell structure, while the latter compresses the network locally by removing unimportant connections in networks with weight-ranking-based pruning. These two methods will separately and sequentially lighten CNN from different scales, raising the efficiency of lightweight CNN design.
- Two lightweight CNNs were built and validated on two typical tasks in the PHM field, fault diagnosis and RUL prediction. Experimental results confirmed the effectiveness and generality of the proposed method.

The rest paper is structured as follows. Section “Principle of cell-based NAS and model pruning” presents the theory of DARTS-based NAS to search for the optimal CNN cell structure and the basic procedure of model pruning. Section “Test benches and datasets” describes two test benches and datasets used for model training and validation. Section “Cell‑based CNN construction for bearing fault classification” presents the cell-based CNN with searched optimal cell to deal with bearing fault diagnosis. Then, Section “Cell-based CNN for bearing RUL prediction” reports the possibility of transferring the cell-based CNN model for classification to realize RUL prediction. Finally, Section “Model pruning for proposed cell-based CNNs” concludes the whole paper.

## Principle of cell-based NAS and model pruning

This section introduces the principle of NAS-based optimal cell exploration and cell-based CNN construction. Moreover, the weights-ranking-based pruning theory and procedure are also briefly described.

### Cell structure and operation definition

**Figure 1:**
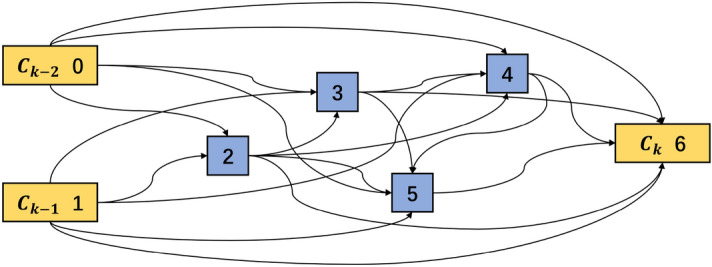
Cell structure with seven nodes.

In the cell-based CNN, a cell is a base in the network that plays a similar role as a layer in the traditional CNN. It is a directed acyclic graph consisting of an ordered sequence of nodes. Each node *x*(*i*) is a latent representation and each directed edge (*i*, *j*) is associated with some operations $o^{(i,j)}$ that transform *x*(*i*). Figure 1 shows an example of a cell with seven nodes, which has two input nodes (nodes 0, 1) and one output node (node 6). Assuming this cell is the kth cell of the whole network, then the input nodes $c_{k-1}$ and $c_{k-2}$ are the outputs from the (k-1)th and (k-2)th cells, respectively, the node $c_k$ is the output node, and the nodes 2, 3, 4, 5 are the intermediate nodes. The edge between two nodes represents a combination of all possible operations, like convolution, separable convolution, dilated convolution, max pooling and average pooling. Each operation contributes to the output of the current node with importance quantified by weights. For each node, the sum of all the operation weights is 1. The cell’s output is obtained by applying a concatenation to all intermediate nodes, and each intermediate node is computed based on its predecessors. Initially, the weights of all operations between nodes are unknown and can be learned during the model training. Therefore, the cell structure searching is equal to learning the operations between nodes.

### Cell structure search with continuous relaxation and optimization

Many different search strategies have been proposed to search for an optimal cell structure, like random search, Bayesian optimization, evolutionary methods, reinforcement learning, and gradient-based methods^23^. However, they are time-consuming. In comparison, DARTS can reduce the training time significantly and is adopted in this study. The essence of the DARTS algorithm is to connect the nth node with all the $n-1$ nodes before it. Then, there are multiple candidate operations between two connected nodes, and each operation is assigned to a corresponding architecture weight for participating in the network learning. Finally, the operation with the highest final architecture weight will be retrained.

Let $x^{j}$ denote the jth intermediate node in the cell, and $o^{(i,j)}$ represents the candidate operation between the ith and jth nodes, with $i < j$. The value of the jth node can be expressed as:

1 $$\begin{aligned} x^{(j)}=\sum _{i<j} o^{(i,j)} \left( x^{(i)} \right) \end{aligned}$$

The candidate operations defined in this study are listed in Table 1, and their set is denoted as ${\mathscr {O}}$.

**Table 1:** Operation set definition.

Number	Operation
1	None
2	Average pooling, filter 3$\times$3
3	Max pooling, filter 3$\times$3
4	Skip connections
5	Separable convolution, filter 3$\times$3
6	Separable convolution, filter 5$\times$5
7	Separable convolution, filter 7$\times$7
8	Dilated convolution, filter 3$\times$3
9	Dilated convolution, filter 5$\times$5
10	Combined convolutions, filter 7$\times$1 and 1$\times$7

Let $\alpha = \lbrace \alpha ^{(i,j)} \rbrace$ be the architecture weight of candidate operation $o^{(i,j)}$ between the ith and jth nodes. Then, the weighted sum of the whole candidate operations can be calculated by Eq. (2), where ${\overline{o}}^{(i,j)}(x)$ stands for the combined operations of candidate operation set *x*.

2 $$\begin{aligned} {\overline{o}}^{(i,j)}(x) = \sum _{o \in {\mathscr {O}}} \alpha _{o}^{(i,j)}o(x) \end{aligned}$$

To make the search space continuous, the softmax function is applied over all possible operations:

3 $$\begin{aligned} {\overline{o}}^{(i,j)}(x) = \sum _{o \in {\mathscr {O}} } \frac{ \textrm{exp}\left( \alpha _o^{(i,j)} \right) }{\sum _{o' \in {\mathscr {O}} } \textrm{exp} \left( \alpha _{o'}^{i,j} \right) } o(x) \end{aligned}$$

Then, the task of cell architecture search reduces to learning a set of continuous weight variables $\alpha = \lbrace \alpha ^{(i,j)} \rbrace$, where $\alpha$ represents the probability distribution of the candidate operations. At the end of search, a discrete architecture can be obtained by replacing each mixed operation ${\overline{o}}^{(i,j)}$ with the most likely operation, i.e., $o^{(i,j)}={\text {argmax}}_{o \in {\mathscr {O}}} \alpha _o^{(i, j)}$. Besides $\alpha$, another parameter ($\omega$) to be learned stands for the weights in the network, such as the weights in convolution and pooling filters. After relaxation, the goal is to jointly learn the architecture $\alpha$ and the weights $\omega$ within all the mixed operations. DARTS aims to find the optimal structure by optimizing the validation loss based on gradient decent. Let ${\mathscr {L}}_{train}$ and ${\mathscr {L}}_{val}$ be the training and the validation loss respectively. Both losses are determined not only by the architecture weights $\alpha$, but also the weights $\omega$. The goal for architecture search is to find $\alpha ^*$ that minimizes the validation loss ${\mathscr {L}}_{val}(\omega^*,\alpha ^*)$, where the weight $\omega^*$ associated with the architecture are obtained by minimizing the training loss $\omega^*={\text {argmin}}_\omega {\mathscr {L}}_{\text {train }}\left( \omega, \alpha ^*\right)$. This implies a bilevel optimization problem with $\alpha$ as the upper-level variable minimizing the validation loss ${\mathscr {L}}_{val}$ and $\omega$ as the lower-level variable minimizing the training loss ${\mathscr {L}}_{train}$:

4 $$\begin{aligned}{} & {} \mathop {\textrm{min}}\limits _{\alpha }~~{\mathscr {L}}_{val}(\omega^{*}(\alpha ),\alpha ) \end{aligned}$$

5 $$\begin{aligned}{} & {} {\mathrm{s.t.}}~~\omega^{*}(\alpha )=\textrm{argmin}_\omega {\mathscr {L}}_{\textrm{train}}(\omega,\alpha ) \end{aligned}$$

To speed up the optimization process, the architecture gradient can be approximated as follows:

6 $$\begin{aligned} &\nabla _{\alpha } {\mathscr {L}}_{\text {val }}\left( \omega^{*}(\alpha ), \alpha \right) \, \approx \nabla _{\alpha } {\mathscr {L}}_{\text {val }}\left( \omega-\xi \nabla _{\omega} {\mathscr {L}}_{\text {train }}(\omega, \alpha ), \alpha \right) \end{aligned}$$

where $\omega$ denotes the current weights maintained by the algorithm, $\omega^{*}(\alpha )$ means the optimal weight $\omega^{*}$ under given structure $\alpha$, and $\xi$ is the learning rate. Applying chain rule to the approximate architecture gradient, Eq. (6) can be expanded with Eq. (7), where $\omega^{\prime }=\omega-\xi \nabla _{\omega} {\mathscr {L}}_{\text {train }}(\omega, \alpha )$ denotes the weights for the one-step forward model:

7 $$\begin{aligned} \nabla _{\alpha } {\mathscr {L}}_{\text {val }}\left( \omega^{\prime }, \alpha \right) -\xi \nabla _{\alpha , \omega}^{2} {\mathscr {L}}_{\text {train }}(\omega, \alpha ) \nabla _{\omega^{\prime }} {\mathscr {L}}_{\text {val }}\left( \omega^{\prime }, \alpha \right) \end{aligned}$$

The matrix-vector product in the second term in Eq. (7) can be simplified with finite difference approximation as:

8 $$\begin{aligned} \nabla _{\alpha , \omega}^{2} {\mathscr {L}}_{\text {train }}(\omega, \alpha ) \nabla _{\omega^{\prime }} {\mathscr {L}}_{\text {val }}\left( \omega^{\prime }, \alpha \right) \approx \frac{\nabla _{\alpha } {\mathscr {L}}_{\text {train }}\left( \omega^{+}, \alpha \right) -\nabla _{\alpha } {\mathscr {L}}_{\text {train }}\left( \omega^{-}, \alpha \right) }{2 \varepsilon } \end{aligned}$$

where $\varepsilon$ is a small scalar and $\omega^{\pm }=\omega \pm \varepsilon \nabla _{\omega^{\prime }} {\mathscr {L}}_{\text {val }}\left( \omega^{\prime }, \alpha \right)$. This brings complexity reduced from $O(|\alpha \Vert \omega|)$ to $O(|\alpha |+|\omega|)$.

**Figure 2:**
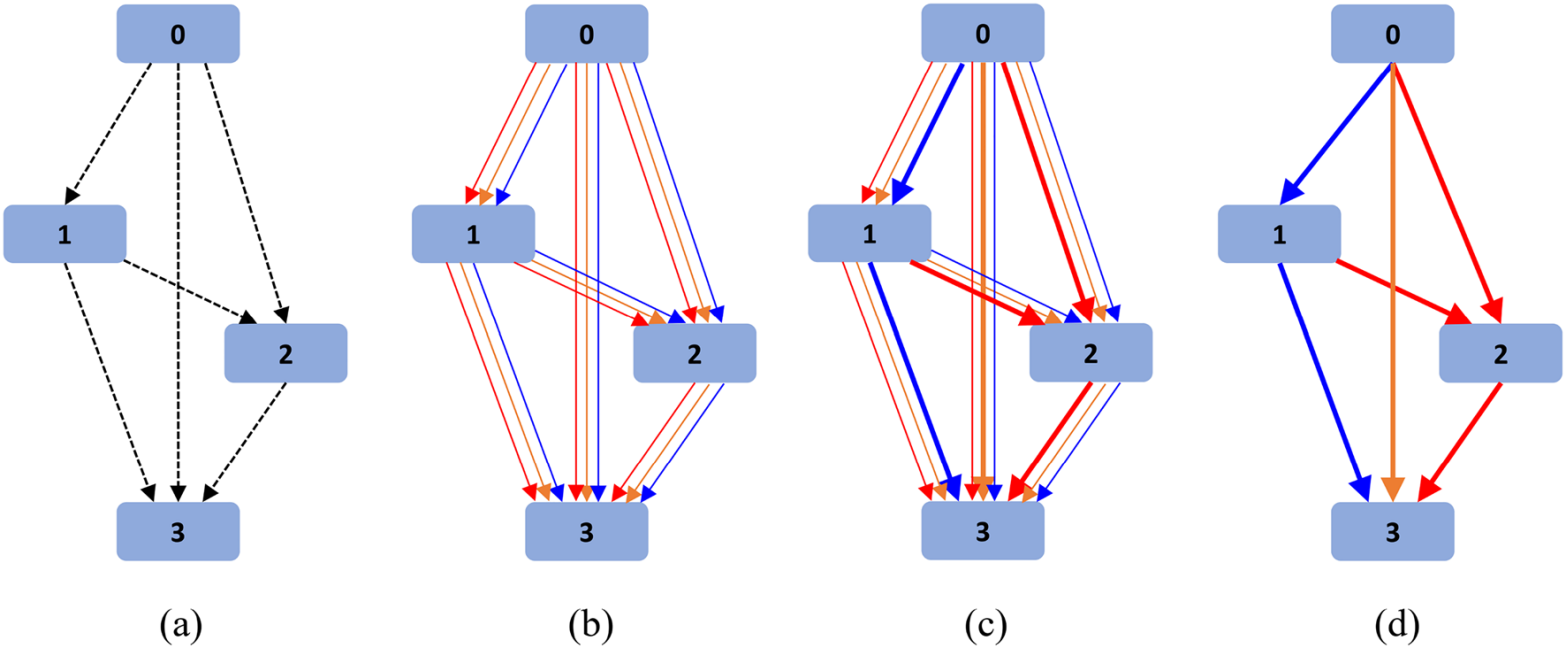
Searching process of cell architecture using DARTS.

For intuitive explanation, Fig. 2 gives an overview of how DARTS searches for the optimal cell structure. At first, as shown in Fig. 2a, the connection $o^{(i,j)}$ between any two nodes *i* and *j* is unknown and assumed to contain all the possible operations defined in the search space. For simplicity, three operations from the operation set are selected for demonstration. The parallel lines in Fig. 2b represent the possible connections, and each represents a kind of operation. They all contribute to the output of the corresponding node, but their importance is different and characterized by the architecture weights. In the search phase, the weights can be optimized by backpropagation. As shown in Fig. 2c, the connections with bigger weights are represented with wider lines. In the end, as shown in Fig. 2d, only the connections with bigger weights are reserved, and the optimal cell structure is obtained.

### From cell to cell-based CNN

**Figure 3:**
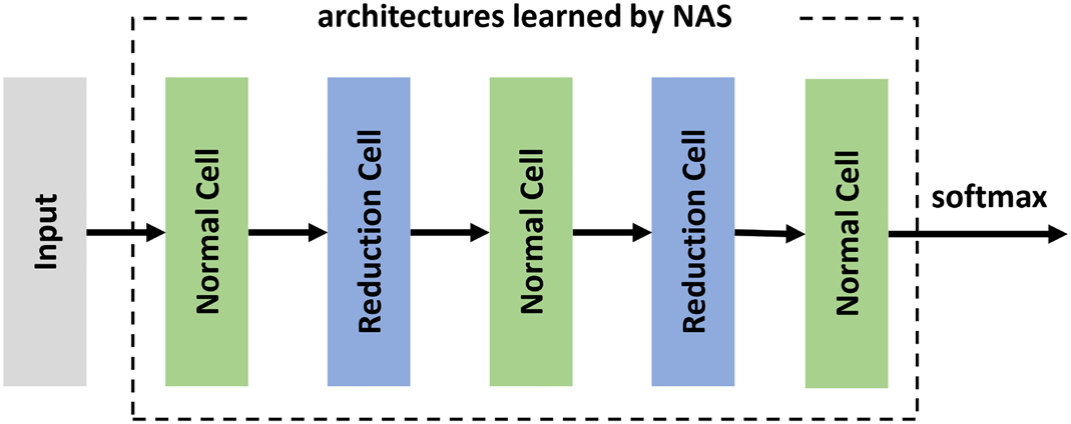
CNN structure with five cells for bearing fault diagnosis.

After the optimal architecture has been determined, the cells can be stacked into a deeper network. Like the convolution layer and pooling layer in general layer-based CNN, two kinds of cells appear alternately in the cell-based network, namely the normal cell and the reduction cell. The normal cells do not alter the size of input feature maps, while the reduction cells reduce the input length and width. As an example, Fig. 3 shows a cell-based network with three normal cells and two reduction cells.

### Weights-ranking-based model pruning

**Figure 4:**
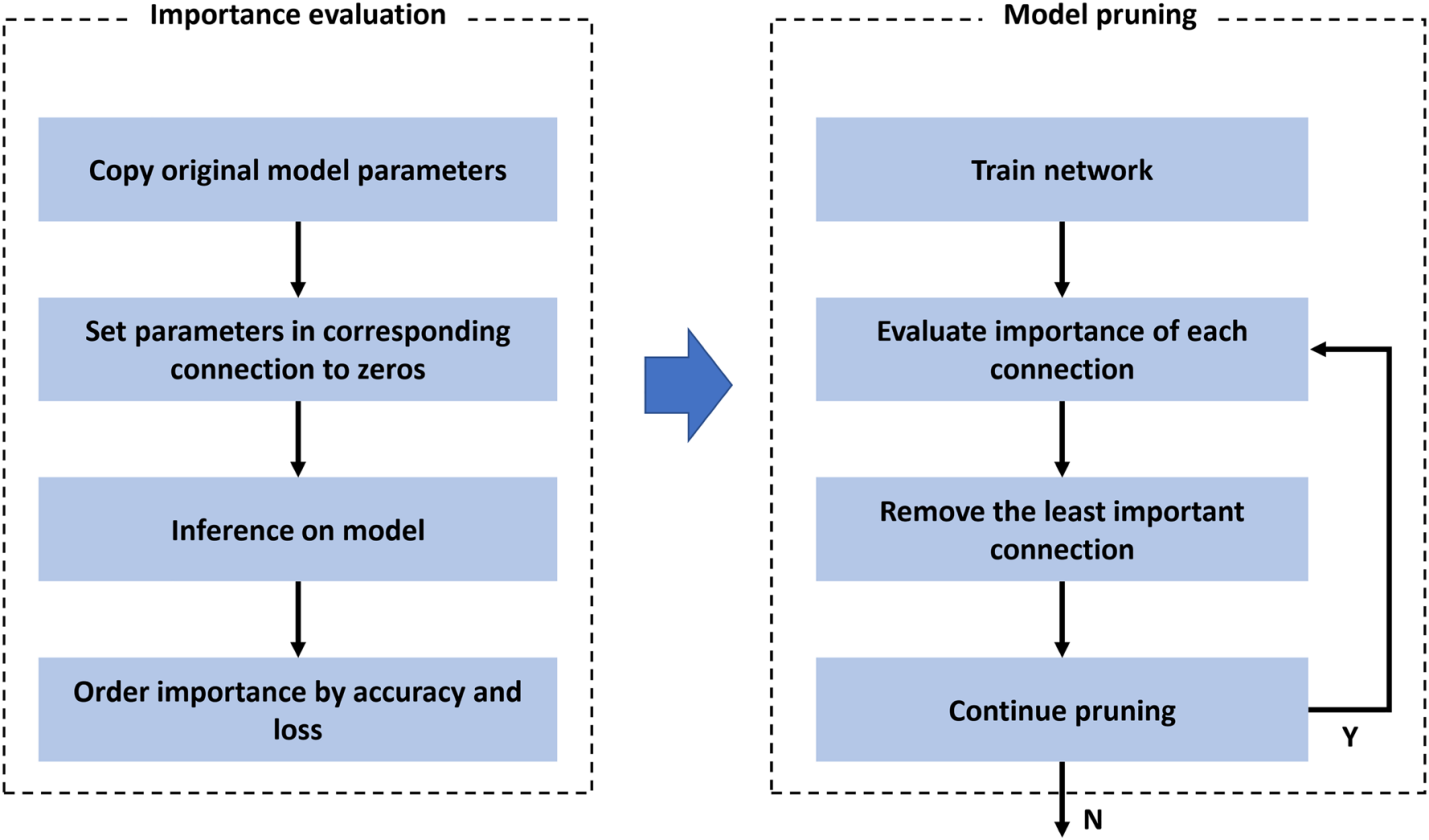
Overview of pruning process for cell-based model.

As mentioned in the introduction, many parameters in a network are redundant and do not contribute much to the output. Thus, if all the neurons in a network can be ranked according to their contributions to performance evaluation metrics, the low-ranking neurons can be removed, resulting in a smaller and faster network. Concerning the development of a light cell-based network, on the one hand, the network size can be reduced by limiting the number of cells and the number of nodes in a cell; on the other hand, the network can be pruned from the aspect of connections by removing less contributory ones. An overview of the connection pruning is shown in Fig. 4. First, the importance of each connection is evaluated, and the least important connection is then removed. The pruning process stops when the boundary condition of model size or accuracy is reached. The boundary condition of model size is determined based on the required CNN model’s final performance rather than the direct storage size. For fault classification, the final performance is identified by fault classification accuracy. While for RUL prediction, it is Mean Squared Error (MSE) loss.

## Test benches and datasets

**Figure 5:**
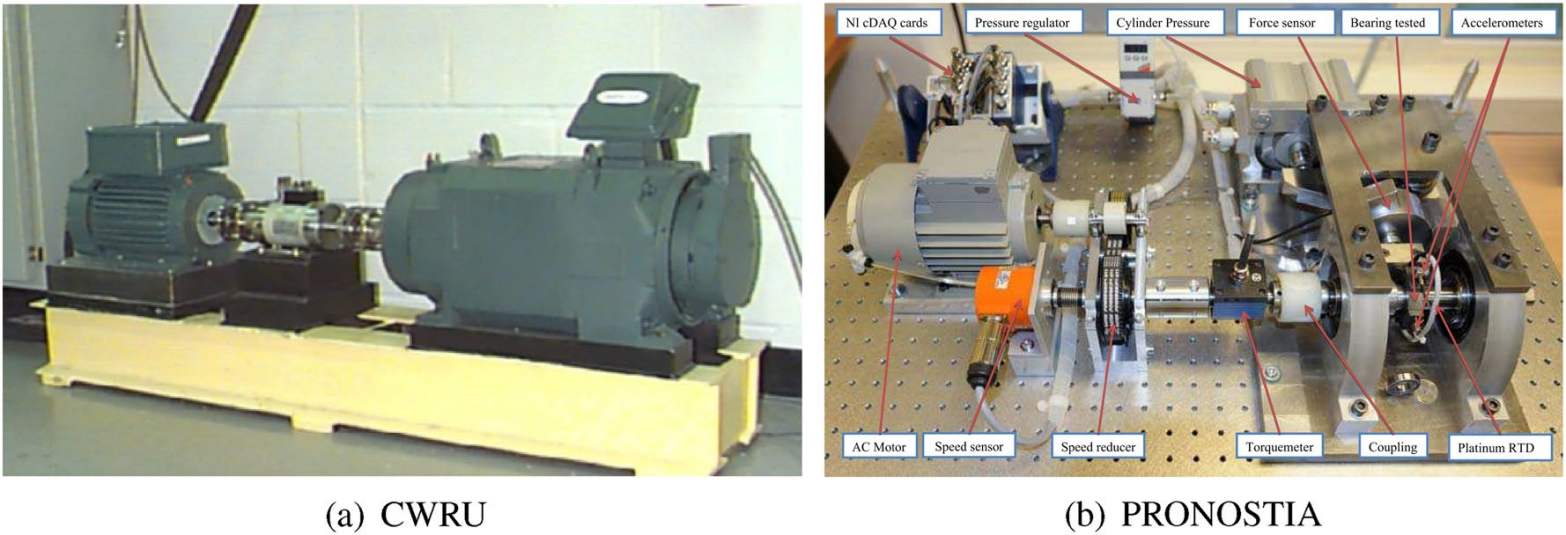
Bearing test benches: CWRU for fault classification (**a**) and PRONOSTIA for RUL prediction (**b**).

The bearing experimental data for fault classification is taken from the Case Western Reserve University (CWRU) bearing dataset^27^. The test bench shown in Fig. 5a comprises of a motor, a dynamometer, control electronics, and test bearings supporting the motor shaft. In addition, accelerometers are attached to the housing to collect the vibration data. Besides normal bearings, there are three failure types: ball fault, inner race fault and outer race fault. Each failure type has three different fault diameters (0.007 inches, 0.014 inches and 0.021 inches) and four different load states (0 HP (horsepower), 1 HP, 2 HP and 3 HP), bringing a total of ten types of bearing conditions. The data collected from the drive end with a sampling frequency of 12 kHz is used in this study for fault classification. With more possible spatial connections between data points, the 2-D CNN contains more information than the 1-D CNN, and thus the 2-D CNN is adopted in this study. Initially, the original acceleration measurement is cut into segments with each length of 4032 and then reshaped into image data with the shape of 3$\times$28$\times$48. This means that the acceleration series are sliced into segments of the same length and then stacked row by row to build a 2-D matrix. In each sample, there are a total of 4032 points of data. The size of the 2-D matrix is defined as 28 $\times$ 48. Therefore, one complete sample can be divided into three such 2-D matrixes. Then, these three 2-D matrixes are combined together and fed into CNN as an image. The ten classes of bearing conditions are labeled with numbers from 0 to 9, with labels 0 for normal condition, 1, 2 and 3 for ball faults, 4, 5 and 6 for inner race faults, and 7, 8 and 9 for outer race faults. Under each fault position, three labels characterizing with increasing numerals stand for the fault size growing from 0.007 to 0.014 and 0.021 inches. For example, label 1 stands for the class of ball fault with a size of 0.007 inches and label 2 for the ball fault with a size of 0.014 inches. The dataset is split into training, validation and test datasets in a ratio of 7:2:1. The dataset description is given in Table 2.

**Table 2:** CWRU bearing data description.

Bearing condition	Number of total samples	N. of samples in training set	N. of samples in validation set	N. of samples in test set	Label
Normal	2401	1183	506	712	0
Ball fault	2822	1397	585	840	1, 2, 3
Inner race fault	2822	1398	574	850	4, 5, 6
Outer race fault	2589	1232	568	789	7, 8, 9

Regarding RUL prediction, the experimental data from the PRONOSTIA platform^28^ is adopted. As shown in Fig. 5b, this test bench operates under three operating conditions with different rotating speeds and radial loads. Table 3 lists the working conditions that have full sensor recordings of temperature and acceleration signals, where $\omega _s$ stands for the shaft rotation speed and $F_r$ the radial load force. Three run-to-failure datasets labeled as *bearing 1_1*, *bearing 2_1* and *bearing 3_1* are applied for model training, while the others are used for the model test. Instead of actual measurement, 38 features in Table 4 are extracted from the time and frequency domains for RUL prediction. The definition of time domain features can be found in^29^. The frequency domain features are derived from the fault characteristic frequencies, with BPFO (Ball Passing Frequency of Outer race) for the outer race fault and BPFI (Ball Passing Frequency of Inner race) for the inner race fault. Take the inner race fault as an example, $A_m^{BPFI_i}{(t)}$ is the amplitude of the ith order of BPFI at time *t*, $\sum _{i=1}^{3}A_m^{BPFI_i}(t)$ is the sum of amplitudes from the first three orders ($i=1,2,3$) of BPFI at time *t*, $\sum _{t=0}^{t}\sum _{i=1}^{3}A_m^{BPFI_i}(t)$ sum of amplitudes from the first three orders ($i=1,2,3$) of BPFI from the beginning ($t=0$) to time *t*. It is the same case with the outer race fault. After the features have been extracted, all the feature data and the output RUL are rescaled by standard normalization before training and testing.

**Table 3:** Dataset description for PRONOSTIA test bench.

	Condition 1	Condition 2	Condition 3
Datasets	$\omega _s$ = 1800 rpm	$\omega _s$ = 1650 rpm	$\omega _s$ = 1500 rpm
	$F_r$ = 4000 N	$F_r$ = 4200 N	$F_r$ = 5000 N
Learning sets	Bearing 1_1	Bearing 2_1	Bearing 3_1
Test sets	Bearing 1_4	Bearing 2_4	Bearing 3_3
	Bearing 1_5	Bearing 2_5	
	Bearing 1_6	Bearing 2_7

**Table 4:** Extracted features for bearing RUL prediction.

Number	Time domain features	Number	Frequency domain features
1–3	Mean	19–24	$A_m^{BPFO_i}{(t)}$ (i = 1,2,3)
4–6	Standard deviation	25–30	$A_m^{BPFI_i}{(t)}$ (i = 1,2,3)
7–9	Sknewness	31–32	$\sum _{i=1}^{3}A_m^{BPFI_i}(t)$
10–12	Kurtosis	33–34	$\sum _{i=1}^{3}A_m^{BPFO_i}(t)$
13–15	Peak-to-peak value	35–36	$\sum _{t=0}^{t}\sum _{i=1}^{3}A_m^{BPFI_i}(t)$
16–18	Root mean square	37–38	$\sum _{t=0}^{t}\sum _{i=1}^{3}A_m^{BPFO_i}(t)$

## Cell-based CNN construction for bearing fault classification

After the theory introduction of the cell structure and cell-based CNN, a cell-based CNN will be built in this section for bearing fault classification. According to the aforementioned theory, two main steps are necessary. One is to search for the optimal cell structure, and another is to build a cell-based CNN with the searched optimal cells and apply it in bearing fault classification.

### Optimal cell searching with base CNN model

#### Base CNN model construction and training

A base CNN model is built at first to implement optimal cell searching. As shown in Fig. 1, the basic CNN consists of three normal cells and two reduction cells, with a softmax function at the end for classification. The cross-entropy is defined as the loss function, and Table 5 summarizes the hyperparameters.

**Table 5:** Hyperparameters setting for base CNN model training.

Name	Value
Batch size	36
Training epochs	20
$\omega$ Learning rate	0.025
$\omega$ Learning rate decay	0.001
$\omega$ Learning rate momentum	0.9
Cells count	5
Random seed	2
$\alpha$ Grad clip	5
$\alpha$ Learning rate	3e−4

**Figure 6:**
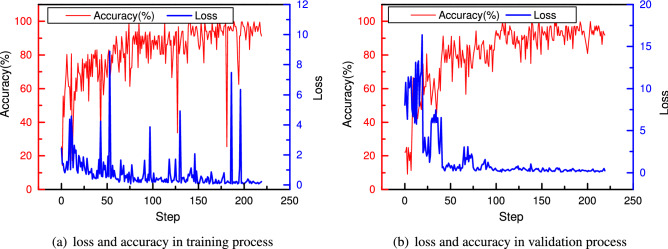
Loss and accuracy in the training process (**a**) and validation process (**b**) in the search phase.

The cell structure contains many edges between nodes, and the entire model is very large, so the model training process requires a large amount of GPU memory. Therefore, the training process is accomplished on a cloud GPU server Tesla V100, and it takes 30 minutes to train the network on a single Tesla V100 GPU for one epoch. Due to the considerable computation, only one-tenth of the whole dataset is used to accelerate the training process. The training and validation results are given in Fig. 6, and it can be found that the training and validation losses decrease continuously at first. However, after 20 steps, the training and validation losses increase violently, and the accuracy decreases from 80% to 60%, which can be explained by the varying cell structure during training. The same phenomenon also happens at the 40th step, 60th step, 100th step, 140th step, 180th step and 195th step.

**Figure 7:**
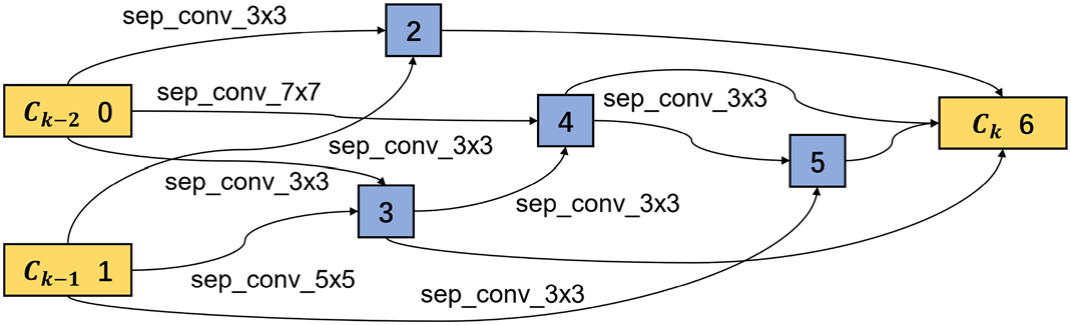
Structure of learned normal cell after 19 steps.

**Figure 8:**
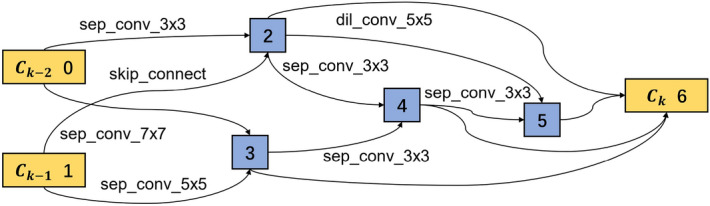
Structure of learned normal cell after 20 steps.

To track how the cell structure changes during the training process, Figs. 7 and 8 present the learned normal cell structures at the 19th and 20th steps, where only two connections with the maximal probability are reserved. We can find that after 19 steps, the separable convolution of $3\times 3$ dominates the operations between node 1 and node 2. However, after 20 steps, the operation with the biggest weight becomes the separable convolution of $7 \times 7$. Because there is a significant change in the structure, most weights in the convolution filters need to be trained again. Therefore, there occurs a drop in the model’s performance accordingly. After training the whole model for 80 steps (8 epochs), it reaches a training accuracy of more than 90% and a validation accuracy of 88%. After training for 150 steps (15 epochs), the training and validation accuracies reach 95%, and the loss rarely changes, indicating that the model is not overfitted and has a good classification performance. Finally, the CNN after training is saved as the base CNN model, from which the optimal cells will be extracted to establish cell-based CNN in the next.

#### Optimal cell structure extraction

**Figure 9:**
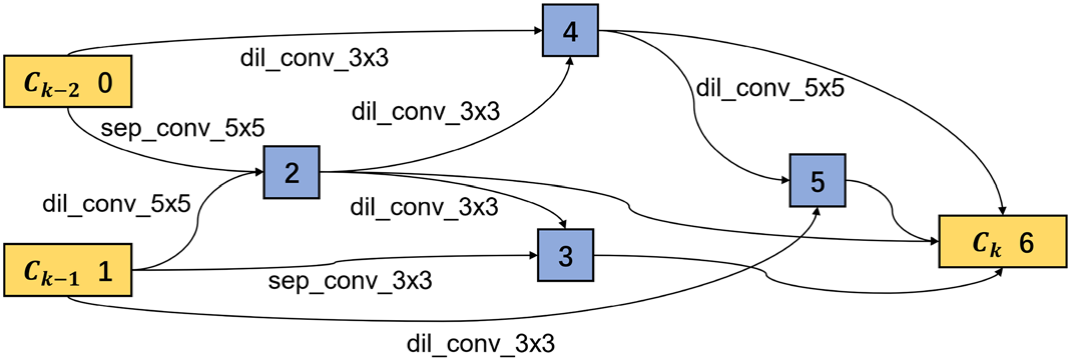
Learned normal cell structure in the search phase.

**Figure 10:**
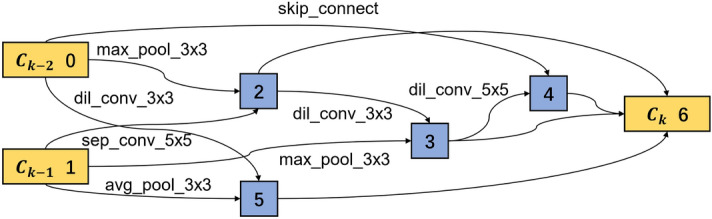
Learned reduction cell structure in the search phase.

The architecture weights $\alpha$ and network weights $\omega$ are optimized in the base CNN model training. Within a single cell, there are several connections between two nodes. Only two connections with the biggest probability are reserved in this study, while the others are discarded. As introduced in Eq. (2), the dominated operation between node *i* and node *j* can be obtained by $\textrm{argmax}_{o\in {\mathscr {O}}} \alpha _{o}^{(i,j)}$. As all the normal cells share the same structure and all the reduction cells share another structure, two kinds of cell structures are extracted. The extracted optimal normal and reduction cells are shown in Figs. 9 and 10 respectively. The normal and reduction cells have seven nodes, but their reserved operations differ. All the input operations of the intermediate nodes are convolution in a normal cell, while a max-pooling operation exists in the reduction cell.

### Cell-based CNN with optimal cells and application in bearing fault diagnosis

After the optimal cell structure has been obtained, a new cell-based CNN with the obtained optimal cells can be built and trained to realize fault diagnosis. In this study, six optimal cells are stacked to construct the CNN, with three reduction cells and three normal cells appearing alternately. Then, the learned network weights $\omega$ in the optimal cells are discarded and trained again, while the operation types and architecture weights are kept. Table 6 gives the parameter set for the optimal network training. Since no architecture weights need to be optimized, a bigger batch size is defined to speed up the training process.

**Table 6:** Hyperparameters setting for optimal CNN training.

Name	Value
Batch size	192
Training epochs	10
$\omega$ Learning rate	0.025
$\omega$ Learning rate decay	0.001
$\omega$ Momentum	0.9
Cells count	6
Random seed	2
$\alpha$ Grad clip	5
$\alpha$ Learning rate	3e–4

**Figure 11:**
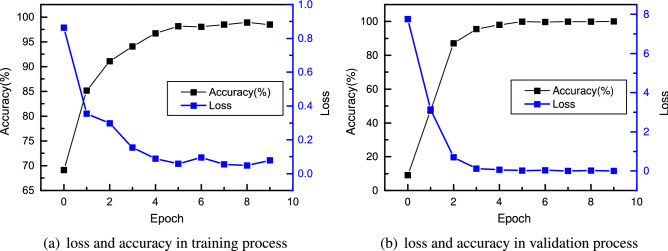
Loss and accuracy of CNN with six cells in the training process (**a**) and validation process (**b**).

**Figure 12:**
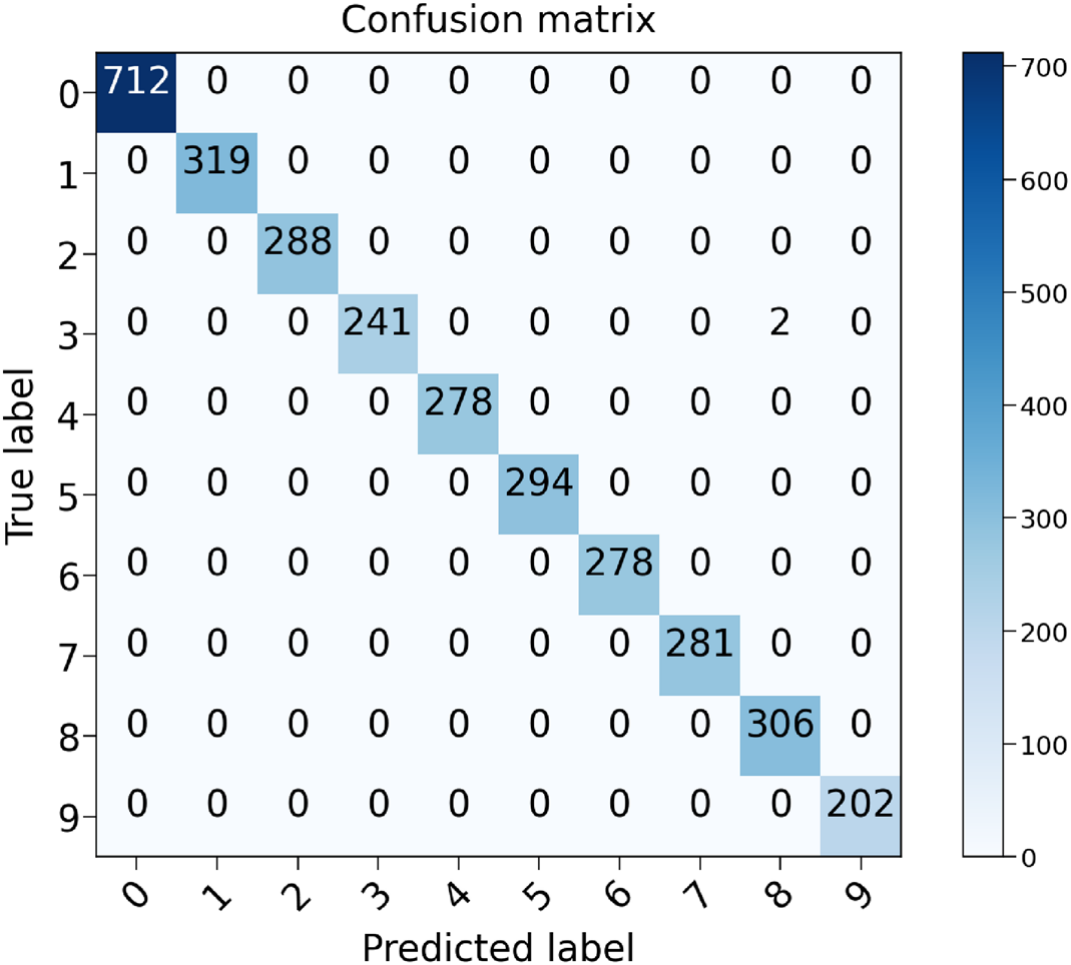
Test confusion matrix of CNN with six cells.

The training and validation results are shown in Fig. 11. After six epochs, the training and validation accuracies reach 98%. It is a fast convergence because the learned optimal cell structure is very efficient in extracting features. The model is then performed on the testing set, and the result is shown in Fig. 12. It can be seen that all the samples in the testing set are correctly classified except two samples in class 3 (ball fault with fault size of 0021 inches), which confirms the excellent performance of the CNN consisting of the learned optimal cells.

### Comparison and discussion of CNN performance with different numbers of cells

**Figure 13:**
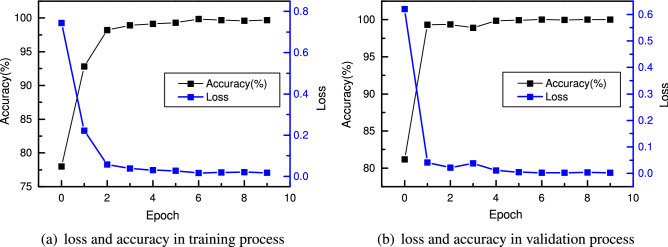
Loss and accuracy of CNN with two cells in the training process (**a**) and validation process (**b**).

The results above show that the optimal cell structure searched by NAS is very efficient in extracting features. The CNN model built with six cells has an extremely high test accuracy. In this section, CNNs with two, three, four and five cells are built and compared to study the influence of the number of cells on network performance. First, the network with two cells (one normal and one reduction cell) is trained. The results of training and validation are shown in Fig. 13. Compared to Fig. 11, CNN with only two cells shows a faster convergence. After two epochs, it reaches a training accuracy of 97% and a validation accuracy of 98.6%, while CNN with six cells only achieves a training accuracy of 91%. In the end, it achieves a test accuracy of 99.97%, and only one sample is not correctly classified.

The accuracy and parameter size of CNN with from two to six cells are given in Table 7. A model with two cells only has 0.197MB parameters but achieves an equivalent performance as the other networks with more cells. The models with three and five cells can achieve a test accuracy of 100%. The training accuracy tends to decrease as the number of cells increases. For example, a model with two cells has a training accuracy of 99.672%, while a model with six cells has 98.491%. Conventionally, the more cells a network has, the more training time it needs. Since more cells mean more filter parameters, it needs more training time to learn the parameters. Compared with the base CNN obtained at the cell searching phase, the CNN with two cells has much less parameter size but higher training and test accuracy. In short, the network with two cells has fewer parameters for this classification task but achieves comparable performance.

**Table 7:** Comparison of CNNs with the different number of cells.

Model	Parameter size (MB)	Training accuracy (%)	Validation accuracy (%)	Test accuracy (%)
2 cells	0.197	99.672	99.954	99.969
3 cells	0.232	99.583	99.956	100.000
4 cells	0.589	99.469	99.957	99.968
5 cells	0.706	99.469	99.956	100.000
6 cells	0.737	98.491	100.000	99.937
Base CNN	9.677	98.491	100.000	99.937

## Cell-based CNN for bearing RUL prediction

The cell-based CNN for bearing fault diagnosis has been built and validated in the last section. Generally, the bearing fault classification and RUL prediction are regarded as different tasks and are addressed independently in most cases. In this section, we are trying to use the searched optimal cells from the cell-based CNN for bearing fault diagnosis to build a cell-based CNN for bearing RUL prediction after small modifications.

### Optimal cells structure searching for bearing RUL prediction

**Figure 14:**
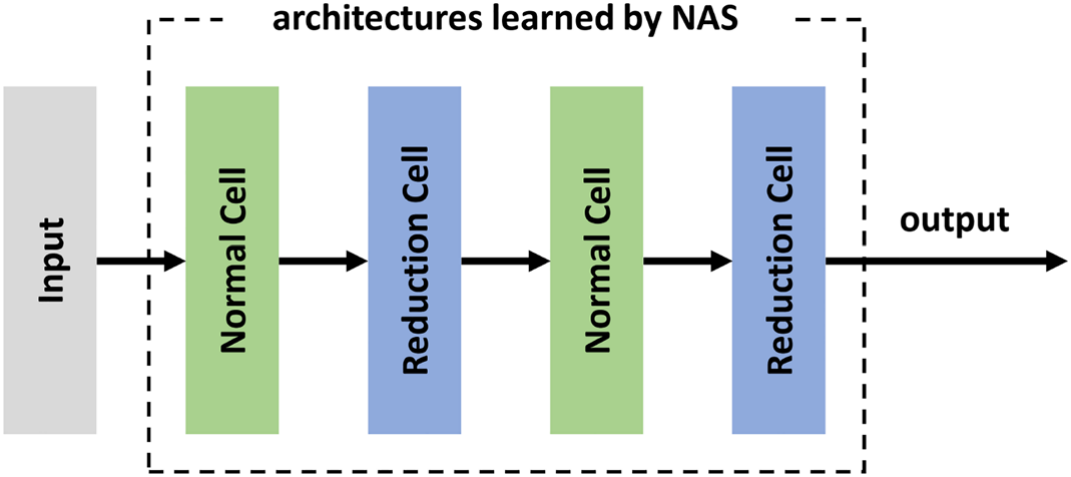
CNN structure with four cells for RUL prediction.

When searching the optimal cells for RUL prediction, different from fault classification, the softmax function is removed, and the cross-entropy loss function is replaced by the MSE. In contrast, the search space is identical to the operations used for fault classification, as defined in Table 1. Additionally, 38 extracted features rather than the original measurements are fed as input. Figure 14 gives the cell-based CNN structure used in the search phase. The entire procedure is the same as used in bearing fault classification. Firstly, an over-parameterized network is built and trained to search for the optimal cell structure. Then, the optimal cells are stacked to build a network that will be trained again to obtain the optimal network.

**Figure 15:**
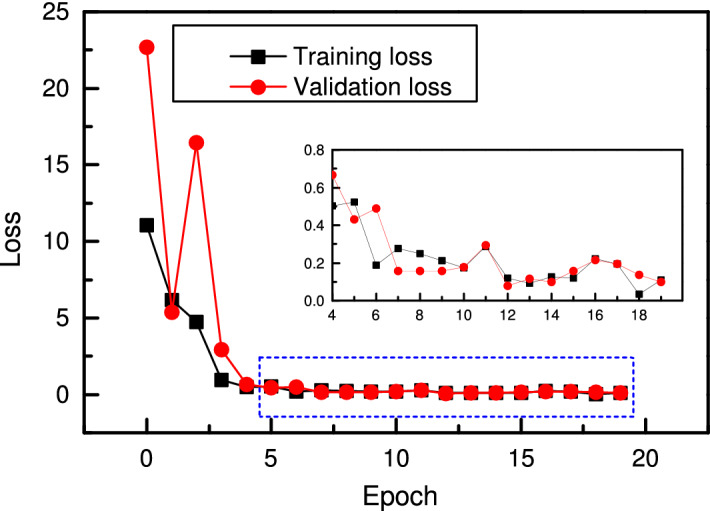
Training loss and validation loss for RUL prediction in the search phase.

**Figure 16:**
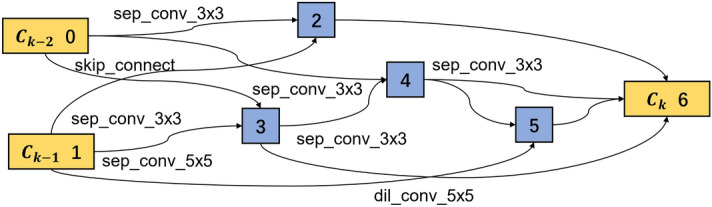
Optimal normal cell structure for RUL prediction.

**Figure 17:**
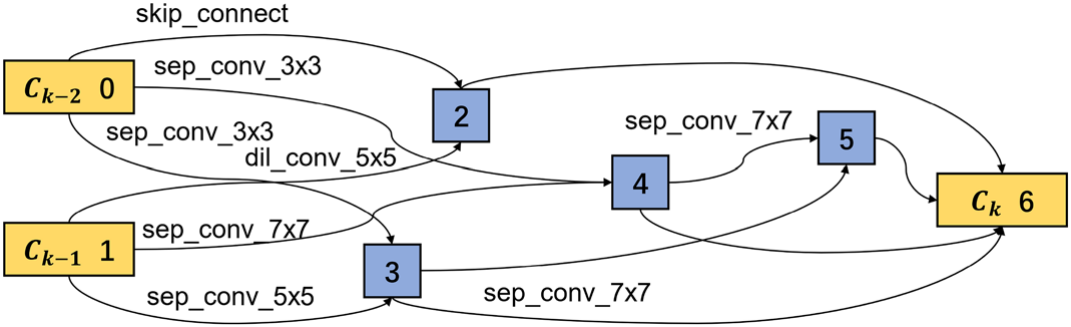
Optimal reduction cell structure for RUL prediction.

There are three working conditions as given in Table 3. Here, condition 1 is taken as an example to explain the entire model-building procedure. Dataset from *bearing 1_1* is used to train the network, and *bearing 1_4*, *bearing 1_5* and *bearing 1_6* are used separately to test the model’s performance. The learning dataset *bearing 1_1* is randomly split into the training and validation datasets with a split ratio of 8:2. The training and validation losses are shown in Fig. 15. In the beginning, the training loss is at a high level. After three epochs, the training loss is 4.66, while the validation loss is 16.61 since the parameters in the model are not fully learned. After five epochs, the training loss decreases to 0.50, and the validation loss decreases to 0.51, proving the training effectiveness. The loss continues to drop when the training goes on. After 20 epochs, the model is trained into convergence. With the same method described in the bearing fault classification, where only two connections with the maximal weights are reserved, the optimal cell structures are extracted as shown in Figs. 16 and 17.

In a normal cell structure, most of the convolution filters are in the size of $3 \times 3$, while in the reduction cell, the filter sizes are bigger. As a result, 25% of the connections are built by filters of $5\times 5$ filters, 37.5% by filters of $7\times 7$ filters and only 25% by filters of $3\times 3$. The main reason is that the bigger convolution filter has a bigger reception field, extracting more global information. In addition, after the reduction cell, the feature map becomes half of the original input. Thus bigger reception field helps to improve the network’s performance.

### Cell-based CNN with optimal cells and application in bearing RUL prediction

**Figure 18:**
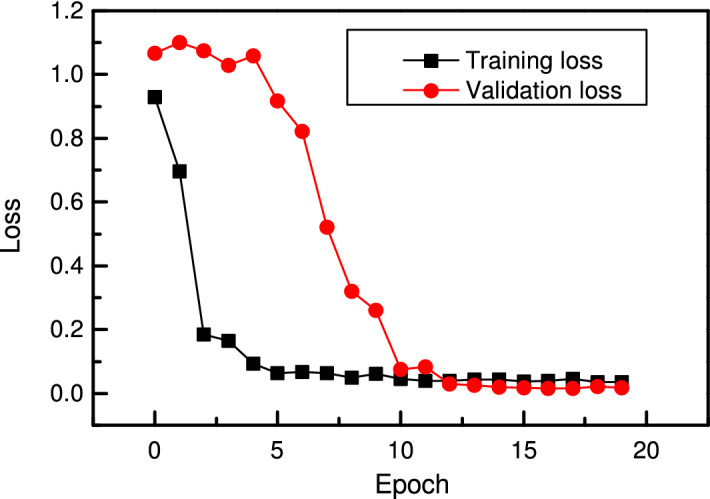
Loss of RUL prediction CNN with four cells in training and validation.

After the optimal cells are extracted, the optimal CNN is then built. The new model for RUL prediction consists of two reduction cells and two normal cells, and its structure is the same as shown in Fig. 14 in the search phase. The optimal cell structure is optimized and fixed in the search phrase, and the operations between nodes are determined. However, their weights need to be trained again before the newly built network is applied to RUL prediction. The optimal CNN is trained for 20 epochs, and the training and validation results are obtained. As shown in Fig. 18, the training loss decreases rapidly in the beginning. It is 0.18 after three epochs and 0.12 after ten epochs. It continues to decrease when the training goes on. Nevertheless, the validation loss remains at a very high level in the beginning and begins to drop after five epochs, from 1.081 to 0.02 in the following ten epochs. After 20 epochs, the training loss decreases to 0.03, and the validation loss decreases to 0.01. The training and validation losses vary in a small range, which indicates that the model is trained into convergence.

**Figure 19:**
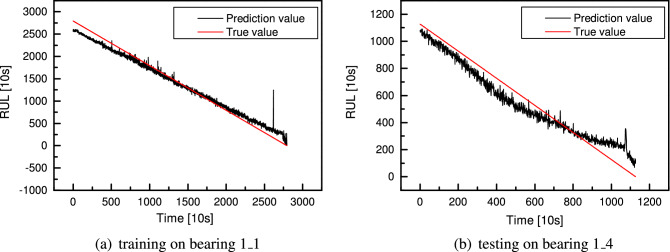
RUL prediction for training *bearing 1_1* (**a**) and testing *bearing 1_4* (**b**) using CNN with four cells.

**Figure 20:**
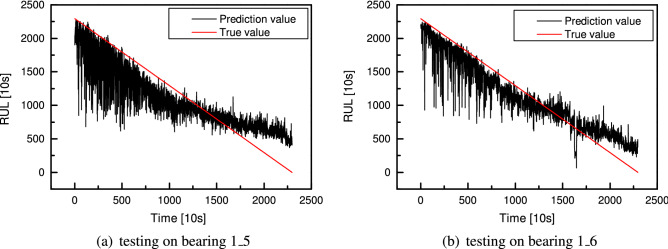
RUL prediction for testing *bearing 1_5* (**a**) and *bearing 1_6* (**b**) using CNN with four cells.

After the CNN with the searched optimal cells has been built and trained, it is applied to the bearing RUL prediction. Since the RUL has been normalized, the predicted data needs to be transformed into the original data range by reverse standard normalization. The model is trained on the *bearing 1_1* dataset, and its performance is tested on the *bearing 1_4*, *bearing 1_5* and *bearing 1_6* datasets. The training result is shown in Fig. 19a, where the x-axis is time, and the y-axis is the remaining useful life with the time unit in 10s. There is a relatively big error at the beginning and end of the entire life, but the predicted error is small in the middle. The test result on *bearing 1_4* shown in Fig. 19b displays a different state. The prediction is accurate in the beginning. As time goes on, the test error first increases and then decreases. In the middle part, there occurs a big error. The actual bearing life varies from 100($\times$10 s) to 50($\times$10 s), while the predicted is about 200($\times$10 s). To evaluate the model’s performance, another criterion R-square is introduced. The R-square of RUL prediction on the training dataset is 0.99, while 0.94 on the test dataset. Though the performance on the test dataset is not as good as on the training dataset, the testing performance is also good, with 94% of the observed RUL samples being explained by the model’s inputs. On the *bearing 1_4* dataset, the actual RUL is 1126($\times$10 s), and the predicted RUL is 1083($\times$10 s), the relative error is 3.8%. The test results on the *bearing 1_5* and *bearing 1_6* are shown in Fig. 20. For the *bearing 1_5*, the actual RUL is 2295($\times$10 s), the predicted RUL is 2065($\times$10 s), and the relative error is 10.0%. As to the *bearing 1_6*, the actual RUL is 2295($\times$10 s), the predicted RUL is 2257($\times$10 s), and the relative error is 1.7%, which is a satisfying result of RUL prediction with experiment data. Moreover, in this study, only one entire RUL dataset is used for training, and the other three are measured under different conditions, making it difficult to generalize. Despite this, the network has a considerable overall performance.

### Comparison and discussion of CNN performance with different numbers of cells

Likewise, to explore the influence of cell count on model performance, the models with two, three, five and six cells are trained separately and compared. To better evaluate the model’s performance with the different number of cells, the training and testing performances of all the models are given in Tables 8 and 9. Comparing all the training results, the models’ R-square is higher than 0.98, indicating that the models have little error during the training and can explain more than 98% of the dataset. Furthermore, the predicted RUL on the training dataset ranges from 2660($\times$10 s) to 3000($\times$10 s), and the actual RUL is 2793($\times$10 s), which confirms the gap between the predicted and actual RULs is small.

**Table 8:** Performance comparion for training *bearing 1_1* using CNN with the different number of cells.

Cell count	Test loss	R-square	Predicted RUL ($\times$10 s)	Actual RUL ($\times$10 s)	RUL error percentage	Training time (s)
2	0.117	0.995	3000	2793	7.40%	168
3	0.229	0.991	2642	2793	5.40%	176
4	0.264	0.989	2660	2793	4.80%	190
5	0.503	0.980	2792	2793	0.04%	193
6	0.185	0.993	2752	2793	1.47%	212

**Table 9:** Comparison of RUL prediction by CNNs with different number of cells.

Cell count	Test condition	Test loss	R-square	Predicted RUL ($\times$10 s)	Actual RUL ($\times$10 s)	RUL error percentage
2	Condition 1_4	0.863	0.965	1281	1126	13.7%
	Condition 1_5	2.704	0.892	2500	2295	8.9%
	Condition 1_6	1.299	0.948	2453	2295	6.8%
3	Condition 1_4	1.574	0.937	1117	1126	0.8%
	Condition 1_5	6.620	0.735	2002	2295	12.7%
	Condition 1_6	3.023	0.879	2161	2295	5.8%
4	Condition 1_4	1.449	0.942	1083	1126	3.8%
	Condition 1_5	5.899	0.764	2066	2295	10.0%
	Condition 1_6	2.673	0.893	2257	2295	1.7%
5	Condition 1_4	1.489	0.940	1159	1126	2.9%
	Condition 1_5	4.414	0.823	2131	2295	7.1%
	Condition 1_6	2.402	0.904	2455	2295	6.9%
6	Condition 1_4	1.228	0.951	1130	1126	0.4%
	Condition 1_5	4.715	0.811	2222	2295	3.2%
	Condition 1_6	2.324	0.907	2292	2295	0.1%

In terms of testing results, as shown in Table 9, all the CNNs can achieve more than 0.9 R-square on *condition 1_4*. Especially the CNN with two cells can even give 0.965 R-square in the test, which is an extraordinary performance. In addition, the predicted RUL is 1281($\times$10 s) and very close to the actual value of 1126($\times$10 s). However, all the CNNs’ performance reduces when tested with data from *condition 1_5*. The models with three and four cells can only achieve R-squares of 0.735 and 0.764, and their relative errors of more than 10% are slightly high. The model with two cells achieves the best R-square of 0.892, and its relative error between the predicted RUL of 2500($\times$10 s) and the actual RUL of 2295($\times$10 s) is 8.9%. As to the test performance under *condition 1_6*, the model with three cells has the smallest R-square of 0.879, while the model with two cells has the biggest R-square of 0.948. Comparing the two cells with six cells, one can find that the former has a relative error of 6.8% while the latter has only a relative error of 0.1%. Although the error percentage of the six-cell model is the smallest among all the models, the overall performance of the two-cell model is better, as it gives the highest R-square and the smallest loss on all the test datasets. The training and testing results on the second and third RUL datasets will not be explained in this paper due to space limitation, but the results of the model with two cells are given in Table 10 as a reference. These results confirm that the cell-based CNN consisting of cells searched by NAS can perform well in both fault diagnosis and RUL prediction.

**Table 10:** Training performance of CNN with 2 cells under conditions 2 and 3.

Condition	Test loss	R-square	Predicted RUL ($\times$10 s)	Actual RUL ($\times$10 s)	RUL error percentage	Training time (s)
Condition 2	0.352	0.986	850	899	5.5%	163
Condition 3	0.996	0.960	508	469	8.3%	151

## Model pruning for proposed cell-based CNNs

Regardless of its growing extraordinary performance, the increasing size of CNN prevents it from being deployed to devices with limited computational resources, like mobile devices and embedded systems. Moreover, CNNs with large capacities usually have significant redundancy among different filters and feature channels. As presented above, the cell-based CNN built with searched optimal cells has a much lighter structure and less parameter size than the traditional CNN. Nevertheless, the proposed cell-based CNN can be further reduced by model pruning, namely by removing the less important connections with minimal loss on final performance.

### Weights-ranking-based pruning

The cells are stacked to build the optimal CNN in this study. The nodes are connected by different operations within a single cell. Thus, there are two ways to reduce the model complexity. Firstly, the number of cells can be controlled during the training phase because the optimal cell structure is already extracted. Secondly, the connections in the cell can be removed if they do not contribute to the model’s performance. As proved in the previous section, after the optimal cell structure has been searched, even a network with only two cells can achieve a test accuracy of more than 99%. Therefore, the number of cells can be decreased to two to get a light cell-based CNN. In the following, this study will focus on pruning connections in the cell, and the weights-ranking-based pruning technology will be adopted.

From the perspective of weights-ranking-based model pruning, a global accuracy list is initialized for saving the model’s results in the beginning. This process is accomplished by setting all the parameters in the connection to zero. For example, the parameters of the first connections are set to zero at the first iteration. Thus, a child model is obtained. Then, the child model’s performance is evaluated by the training dataset. Finally, the accuracy with its corresponding order is saved to the global list. Next, the same task is implemented on the second connection, while the parameters of the first connection are not set to zero and remain the same as their initial values after training. Then the second child model’s accuracy is also obtained. After removing all the connections, the global accuracy list contains all the child models’ accuracy. This list is sorted by accuracy in descending order, where the first item in the list has the highest order, and its corresponding connection is considered the least important one.

### CNN pruning for bearing fault classification

**Figure 21:**
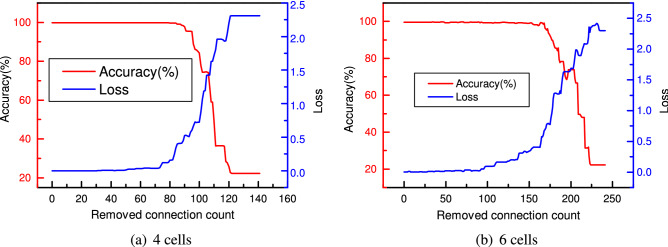
Test loss and accuracy in the pruning of model with four cells (**a**) and six cells (**b**).

Conventionally, the CNN achieves better performance with increased model complexity since it can describe more complex mapping relations from input to output. The optimal cell structure searched with the NAS method is very efficient in extracting features. The CNN with two cells can achieve equivalent performance as the CNN with six cells. Figure 21 shows that the more cells the CNN has, the more unimportant connections can be pruned. The detailed comparison of accuracy and loss is given in Table 11. As revealed, the CNN with two cells maintains the test accuracy of 100% after losing 40% of all connections. The CNN with four cells achieves the same performance after losing 50% of all connections. Although the CNN with six cells cannot achieve a test accuracy of 100% after removing 40% connections, the test accuracy of 99.43% is still extremely high. However, compared with the two-cells CNN, the loss of six-cells CNN is smaller. After 70% of all the connections are pruned, the CNN with two cells still provides an accuracy of 99.69%. In comparison, the CNN with six cells achieves an accuracy of 97.84%. Meanwhile, the CNN with four cells only gets a test accuracy of 95.58%, which is lower than the other two CNNs. After 80% connections are removed, the CNN with two cells still achieves a test accuracy of 90.37%, while the CNN with six cells only gets a test accuracy of 78.31%. Comparing these two CNNs, the CNN with four cells only achieves a test accuracy of 36.48% which is below the industry application requirement. After removing another five percent of the connections, all the CNNs only achieve a test accuracy of less than 75%. Therefore, it can be concluded that the simple and complex CNNs show similar pruning results. Although there are more redundancies in the complex CNN, the percentage of the parameters contributing to the CNN’s performance remains the same level as that of the simple CNN. The complex CNN does not achieve better performance. In this specific case, the CNN with two cells achieves higher test accuracy after training, having higher or equivalent performance after removing the same percentage of the connections.

**Table 11:** Model pruning percentage and accuracy comparison.

Removed connection perentage	2 Cells accuracy (%)	2 Cells loss	4 Cells accuracy (%)	4 Cells loss	6 Cells accuracy (%)	6 Cells loss
10%	100	0.020544	100	0.000849	99.968662	0.003303
20%	100	0.021576	100	0.002730	99.874647	0.011658
30%	100	0.022386	100	0.006277	99.905986	0.011504
40%	100	0.018476	100	0.023563	99.435914	0.050277
50%	99.968662	0.032888	100	0.034999	99.435914	0.161205
60%	99.874647	0.040901	99.780633	0.405087	99.498590	0.310290
70%	99.686619	0.127263	95.581322	0.719031	97.837668	0.410326
80%	90.379191	0.468285	36.477593	1.953201	78.314008	1.272309
85%	72.297086	0.770961	26.073331	2.154107	69.257286	1.632098
90%	22.312755	2.302586	22.312755	2.302586	22.312755	2.302586
100%	22.312755	2.302586	22.312755	2.302586	22.312755	2.302586

### CNN pruning for bearing RUL prediction

**Figure 22:**
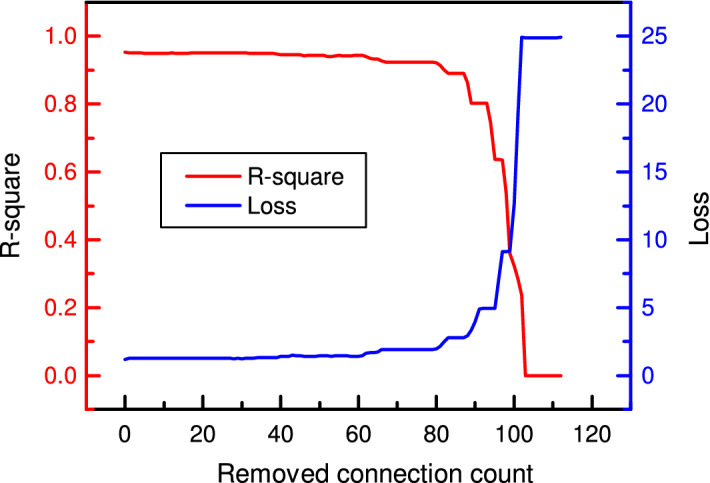
R-square and loss in terms of removed connection count.

**Figure 23:**
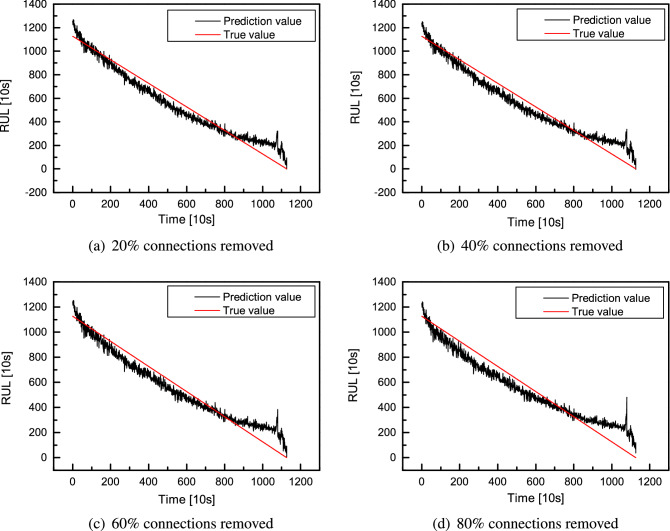
RUL prediction after 20% (**a**), 40% (**b**), 60% (**c**) and 80% (**d**) of connections removed.

Likewise, the cell-based CNN for RUL prediction is also further pruned, and the pruning result is shown in Fig. 22. In the beginning, the training R-square of the model increases to 0.99 after removing the first connection. The high performance remains unchanged after 50% connections have been removed since they are mostly unimportant and contribute less to the model’s performance. After 71 iterations, the R-square drops to under 0.8, and the loss increases to 7.6, indicating a big gap between the predicted and actual values. Then, the slope of the curve begins to increase since there are few connections left in the model. Each further removed connection contributes much to the model’s performance. The pruned model is tested to have a clear look at the model’s performance. Here, the first run-to-failure dataset *bearing 1_1* is selected as the training dataset, and *bearing 1_4* is selected as the testing dataset. The predicted results are transformed into the original RUL scale. The model’s performance after removing 20%, 40%, 60% and 80% of connections is shown in Fig. 23.

Take the CNN with two cells as an example, a comparison of the pruned results is shown in Table 12. The model before pruning gives an R-square of 0.965 on the test dataset, and the RUL prediction error is 13.7%. The connections are removed during pruning, and the R-square drops. It is 0.952 when 20% of connections are removed and further drops to 0.925 when 80% of connections are removed. Meanwhile, the loss increases from 1.206 to 1.874. However, the RUL prediction error is different from the R-square performance during pruning. The predicted RUL is 1281($\times$10 s) before pruning and becomes 1255($\times$10 s) after pruning 20% of connections, and the latter is more close to the actual RUL of 1126($\times$10 s). As a result, the error drops from 13.7% to 11.4%. Comparing the model’s performance during the period from 200($\times$10 s) to 400($\times$10 s), the gap between the actual and predicted values increases when more connections are removed. Since the model’s input has low dimensions, the entire pruning process costs 8 minutes. In the beginning, removing every 20% of connections costs about two minutes. However, as the pruning goes on, there are fewer connections reserved in the model, and the pruning gets faster. Therefore, after removing 60% connections, removing the other 20% of connections costs only ${69}\,\textrm{s}$.

Finally, to verify the superiority of the proposed method, some comparisons with the latest published works are made. For example, in^8^, an integrated multitasking intelligent bearing fault diagnosis scheme was proposed, which can achieve bearing fault detection without any labeled fault data. The fault diagnosis accuracy on CWRU dataset can achieve 96.65% with only 10% of training samples. In contrast, the proposed 4-cells lightweight CNN can yield an accuracy of 99.78% even 60% of the connections have been removed, which indicates the proposed method has a higher fault classification accuracy and a much smaller model size. Regarding RUL prediction on the PRONOSTIA test bench, compared with the graph neural network proposed in^30^, our proposed method exceeds in both R-Squared of RUL prediction and model size, but has weaker interpretability.

**Table 12:** Pruning results comparison for RUL prediction.

Removed connection count	Predicted RUL ($\times$10 s)	RUL error percentage	Loss	R-square	Pruning time costs (s)
0	1281	13.7%	0.863	0.965	0
20	1255	11.4%	1.206	0.952	128
40	1240	10.1%	1.276	0.949	236
60	1242	10.3%	1.426	0.943	325
80	1229	9.1%	1.874	0.925	394

## Conclusion

In recent years, layer-based CNN is becoming more and more complex with continuously growing deeper layers and parameter sizes. Though better performance has been obtained, the complex network structure constrains its application with limited computation and storage sources in the practical industry. This paper proposes a two-step hierarchical method with DARTS-based NAS and model pruning to address this problem. On the one hand, DARTS is applied to search for the optimal cell architecture. After the optimal cell structures are obtained, they are stacked to build the optimal CNN. The number of cells in the CNN is controlled after the influence analysis on CNN’s performance to reduce the network size from the whole. On the other hand, the constructed cell-based CNN is further reduced by removing unimportant connections based on weights-ranking-based pruning, which further compresses the network locally. A light cell-based CNN is obtained through optimization from these two directions. Two validation cases are designed to validate the proposed method. First, the cell-based CNN is validated with the CWRU bearing dataset for the bearing fault classification. It shows a fast convergence during the training and achieves the test accuracy of 99.97% with only two cells. For the bearing RUL prediction, the CNN is performed on the dataset from the PRONOSTIA platform. It also gives an outstanding performance. The CNN with only two cells can achieve a high R-square ranging from 0.892 to 0.965 under different conditions. In terms of model pruning, results show that the CNN with only two cells for fault classification can still reserve a training accuracy of 99% after removing 50% of all the connections. Meanwhile, the CNN model for RUL prediction also gets an R-square of 0.9 after removing 50% connections. In short, with DARTS-based NAS and weights-ranking-based model pruning, an efficient cell-based CNN with a light size and extraordinary performance can be obtained to achieve both fault diagnosis and RUL prediction. It lays the potential to realize light CNN in real-time embedded systems with limited computation and storage sources. With the proposed two-step hierarchical method, a lightweight CNN can be easily and sequentially designed. Though only validated with two typical tasks in the PHM, the proposed method can be extended to other fields and also adapted to other networks like the lightweight LSTM. As to the outlook, research in the future will focus on implementing the proposed light cell-based CNN in embedded systems and testing its performance, such as the FLOPs (floating point operations), inference time, inference complexity, and computing power. Furthermore, how to involve the performance of hardware implementation altogether the accuracy metric when searching for the optimal cell structure also deserves further research. In addition, the hyperparameters optimization in cell searching will be considered, how to transform the pruning as an optimization issue by introducing the $L_1$ or $L_2$ norm will be addressed. New basis cell structures, more kinds of operations within cells, new network structures, and applications on other kinds of networks or more experimental data will also be explored.

## Data Availability

The datasets generated and analyzed during the current study are available in the dropbox repository, https://www.dropbox.com/scl/fo/2cygys8khtyzj3ju7ein2/h?dl=0&rlkey=xpy6kjsicup02dusi7gfg916i.
